# Neutrophil extracellular trap suppression in rheumatoid arthritis patients treated with biologic therapy: a prospective observational study

**DOI:** 10.1007/s10067-026-08454-8

**Published:** 2026-10-03

**Authors:** Georgios V. Papamichail, Akrivi Chrysanthopoulou, Nikolaos A. Georgiadis, Christina A. Kapsali, Vasileios G. Xydis, Athanasios Kitsakos, Alexandros A. Drosos, Paraskevi V. Voulgari

**Affiliations:** 1 Department of Emergency Medicine, Medical School, University of Ioannina, Ioannina, Greece; 2 Laboratory of Molecular Immunology, Department of Biological Applications and Technology, School of Health Sciences, University of Ioannina, Ioannina, Greece; 3 Department of Rheumatology, School of Health Sciences, Faculty of Medicine, Medical School, University of Ioannina, 45110 Ioannina, Greece; 4 Department of Paediatrics, School of Health Sciences, Faculty of Medicine, Medical School, University of Ioannina, Ioannina, Greece; 5 Department of Radiology, Medical School, University of Ioannina, Ioannina, Greece

**Keywords:** Atherosclerosis, Biologic Drugs, Extracellular Traps, Histones, Rheumatoid Arthritis

## Abstract

**Background:**

Rheumatoid arthritis (RA) is associated with increased cardiovascular risk, partly driven by chronic systemic inflammation. Neutrophil extracellular traps (NETs) have emerged as important mechanistic links between sustained immune activation and early vascular injury.

**Objective:**

To evaluate changes in NET-associated activity following initiation of biologic therapy in patients with RA and to investigate the relationship between treatment- associated changes in NETs, lipid profile, and subclinical atherosclerosis.

**Methods:**

In this prospective single-arm observational study, 36 patients with RA initiating biologic therapy were evaluated at baseline and after 6 months of treatment. Disease activity was assessed using DAS28-CRP, C-reactive protein (CRP), and erythrocyte sedimentation rate (ESR). Circulating citrullinated histone H3 (CitH3) levels were measured by ELISA as a robust marker of NETs. Carotid intima-media thickness (cIMT) was assessed using high-resolution B-mode ultrasonography. Linear regression analyses were performed to investigate the relationship between NETs activity, inflammatory burden, lipid profile, and subclinical atherosclerosis, both cross-sectionally and longitudinally following biologic therapy.

**Results:**

Following initiation of biologic therapy, disease activity and inflammatory markers improved significantly. Circulating CitH3 levels decreased from a median 3.89 (0.11–32.78) to 2.56 (0.07–8.39; *p*-value 0.025) while cIMT decreased from a median 1 (0.7–1.6) mm to 0.75 (0.6–1.3 mm; *p*-value < 0.001) after 6 months of treatment. HDL cholesterol increased significantly from 56.36 ± 16.21 to 67.47 ± 17.56 mg/dL (*p* < 0.001). At follow-up, CitH3 levels were independently associated with ESR (β = 0.448, *p* = 0.018), indicating a relationship between NETs and systemic inflammatory burden. Longitudinal analyses demonstrated that changes in HDL cholesterol were independently associated with changes in CitH3 (β =  − 0.505, *p* = 0.003), whereas changes in DAS28, CRP, ESR, oxLDL, and cIMT were not independently associated with ΔCitH3. No significant differences in ΔCitH3 were observed between anti-TNF and non-anti-TNF therapies.

**Conclusions:**

In this prospective observational cohort, initiation of biologic therapy was associated with reduced circulating CitH3 levels, alongside improvements in disease activity, inflammatory markers, HDL cholesterol, and cIMT. The longitudinal association between increases in HDL cholesterol and reductions in CitH3 was independent of changes in disease activity and cIMT, generating a hypothesis regarding a potential immunometabolic relationship between HDL cholesterol and NET-associated activity. These findings should be considered exploratory and require confirmation in larger, controlled prospective studies.

**Table:** 

**Key Points** • *Biologic therapy was associated with a significant reduction in circulating CitH3 levels, consistent with reduced NET-associated activity in patients with RA.* • *Changes in HDL cholesterol independently predict changes in NETs, suggesting a possible HDL–NETs immunometabolic axis in RA patients under biologic treatment.* • *Concurrent improvement in NETs and subclinical atherosclerosis supports a potential role for NETs in RA-associated cardiovascular risk.* • *Similar reductions in CitH3 were observed across biologic treatment classes; however, the small and unequal subgroup sizes limit the ability to draw conclusions regarding class-specific effects.*

**Supplementary Information:**

The online version contains supplementary material available at https://doi.org/10.1007/s10067-026-08454-8.

## Introduction

Rheumatoid arthritis (RA) is a systemic inflammatory disease of unknown etiology that primarily affects the synovial membrane of the joints. The chronic inflammatory process leads to synovial hyperplasia and progressive destruction of cartilage, bone, and ligaments, while multiple extra-articular manifestations contribute to increased morbidity and mortality [1, 2].

The pathogenesis of RA is complex and involves the interplay of genetic, environmental, immunological, and epigenetic factors. A widely accepted hypothesis suggests that a susceptible genetic background, in combination with environmental triggers, leads to the generation of neoepitopes and activation of immune responses, ultimately resulting in chronic synovitis and joint destruction [3].

Neutrophil extracellular traps (NETs) are web-like structures released by activated neutrophils in response to various stimuli. They consist of decondensed chromatin decorated with antimicrobial and cytotoxic proteins, including neutrophil elastase (NE), myeloperoxidase (MPO), histones, and defensins [4–6]. Emerging evidence suggests that NETs play a key role in the pathogenesis of RA, as increased NET formation has been observed in both peripheral blood and synovial tissue of RA patients. NETs contribute to autoimmunity by promoting protein citrullination and the generation of anti-citrullinated protein antibodies (ACPAs), while also amplifying inflammatory responses within the synovium [7].

In parallel, increasing attention has been given to the role of release of NETs in atherosclerosis. Atherosclerosis is now recognized as a chronic inflammatory disease, with inflammation contributing to all stages of plaque development and progression [8–10]. Patients with RA exhibit an increased burden of subclinical atherosclerosis, which may improve following effective anti-inflammatory treatment [11, 12].

Biologic therapies have revolutionized the management of RA by targeting key inflammatory pathways. However, their effect on NETs as well as the potential links between NET formation, disease activity, and vascular damage, remain incompletely understood.

The aim of this study was to investigate the impact of biologic therapy on NETs in patients with RA and to evaluate its association with disease activity, lipids and subclinical atherosclerosis.

## Methods

### Study design and participants

This prospective longitudinal observational study was conducted at a single tertiary University Center between March 2023 and March 2026. Among 773 patients with RA followed at the Rheumatology Clinic of the University Hospital of Ioannina, Greece, 36 patients fulfilled the inclusion criteria and were enrolled. Eligible patients met the following inclusion criteria: age between 18 and 75 years, fulfillment of the 2010 ACR/EULAR classification criteria for RA [13], and an active disease status requiring the initiation of biologic disease-modifying antirheumatic drugs (bDMARDs). At entry, all patients were receiving stable baseline doses of conventional synthetic DMARDs (csDMARDs), predominantly methotrexate or leflunomide and prednisolone 5 mg daily.

To isolate the specific relationship between inflammation-driven NET formation, lipid profile and vascular remodeling, a stringent set of exclusion criteria was applied to filter out confounding traditional cardiovascular factors. Patients were excluded if they presented with a personal or family history of coronary artery disease; current or past smoking; valvular heart defects; chronic infectious diseases; thyroid disorders; diabetes mellitus; active malignancy; chronic kidney disease; hypertension; or any form of dyslipidemia/metabolic syndrome, including anyone receiving lipid-lowering agents or agents that may alter levels of lipids.

All participants underwent synchronized clinical, laboratory, and carotid imaging assessments at two designated time points: at baseline (immediately prior to starting biologic therapy) and at a 6-month follow-up. Written informed consent was secured from all subjects. The study was approved by the Institutional Ethics Committee of the University Hospital of Ioannina (Approval No. 6/11–03–2025) and adhered fully to the principles of the Declaration of Helsinki.

### Clinical assessment

All patients underwent a comprehensive clinical evaluation at baseline and at follow-up after initiation of biologic therapy. Demographic characteristics, disease duration, disease activity score (DAS28), and current treatments were recorded at both time points [14] and are presented in Table 1.

**Table 1:** Clinical, inflammatory, lipid, and vascular characteristics at baseline and after 6 months of biologic therapy (*n* = 36)

Characteristic	Baseline	6-month follow-up	*p*-value
Age (years)	58.7 ± 11.5	–	–
Female sex, n (%)	28 (77.8)	–	–
Male sex, n (%) Disease duration (years)	8 (22.2) 5.2 ± 1.2	–	–
IgM RF positive, n (%)	30 (83.3)	–	–
ACPA positive, n (%)	24 (66.6)	–	–
Methotrexate use, n (%)	30 (83.3)	–	–
Leflunomide use, n (%)	6 (16.7)	–	–
Anti-TNF therapy, n (%)	23 (63.9)	–	–
Prednisolone use, n (%)	36 (100)	–	–
Prednisolone dose (mg/day)	5	–	–
Total cholesterol (mg/dL)	214.08 ± 60.26	228.97 ± 46.90	0.085
Triglycerides (mg/dL)	133 (54–301)	123 (58–323)	0.928
HDL (mg/dL)	56.36 ± 16.21	67.47 ± 17.56	< 0.001
LDL-C (mg/dL)	138.28 ± 43.91	132.69 ± 37.46	0.277
CRP (mg/L)	6 (1–51)	3 (1–12)	< 0.001
ESR (mm/h)	27.5 (9–78)	13.5 (2–45)	< 0.001
DAS28	3.59 (2.03–6.8)	2.97 (1.59–6.56)	< 0.001
cIMT (mm)	1 (0.7–1.6)	0.75 (0.6–1.3)	< 0.001
CitH3 (ng/mL)	3.89 (0.11–32.78)	2.56 (0.07–8.39)	0.025

Comparison over time was made by applying Paired sample t-test or Wilcoxon signed rank test as appropriate. Results are expressed in mean ± SD or median (Min–Max), unless otherwise noted

*AU* Absorbance Units, *ACPA +*  positivity for anti-citrullinated protein antibodies, *cIMT* carotid artery intima-media thickness, *CitH3* citrullinated histone H3, *CRP* C-reactive protein, *DAS28* disease activity score 28 joint, *ESR* erythrocyte sedimentation rate, *HDL-C* high density lipoprotein cholesterol, *LDL-C* low density lipoprotein cholesterol, *LFN* leflunomide, *MTX* methotrexate, *PDN* prednisone, *RF +*  rheumatoid factor positivity, *TC* total cholesterol, *TGs* triglycerides

### Laboratory monitoring

Blood samples were obtained at baseline and at follow-up after initiation of biologic therapy for laboratory analysis. C-reactive protein was measured by nephelometry, and ESR was determined using an automated analyzer. IgM rheumatoid factor (IgM RF) was measured using Latex Agglutination, anti-citrullinated protein antibodies* (*ACPA) using ELISA (AESKULISA CCP, AESKU Diagnostics) and antinuclear antibodies (ANA) using indirect immunofluorescence.

### Serum collection and CitH3 quantification

Peripheral venous blood was drawn into SST II Advance serum separator tubes (Becton, Dickinson and Company, Franklin Lakes, NJ, USA). Samples were allowed to clot at room temperature according to manufacturer’s recommendations and were then centrifuged at 2,000 × g for 10 min. The resulting serum was aliquoted into low-binding polypropylene tubes and stored at −  80 °C until analysis, avoiding repeated freeze–thaw cycles.

Serum levels of CitH3, a validated surrogate marker of NET formation, were quantified using a commercially available ELISA kit (Cayman Chemical, Ann Arbor, MI, USA), following the manufacturer’s instructions. Briefly, serum samples were equilibrated on ice, diluted in assay buffer as specified, and loaded in duplicate together with the provided standards onto precoated 96-well plates. After a 2-h incubation at room temperature, plates were washed five times with 1 × wash buffer, followed by a 1-h incubation with horseradish peroxidase (HRP)-conjugated monoclonal detection antibody. Plates were washed again, tetramethylbenzidine (TMB) substrate was added for 30 min in the dark, and the reaction was stopped using the supplied stop solution. Absorbance was measured at 450 nm using a microplate reader. According to the manufacturer’s performance characteristics, the intra-assay coefficients of variation (CVs) were 3.6%, 8.8%, and 19.9% at CitH3 concentrations of 4.6, 0.9, and 0.4 ng/mL, respectively, while the inter-assay CVs were 6.0%, 10.9%, and 29.7% at concentrations of 4.6, 0.9, and 0.3 ng/mL, respectively.

### Subclinical atherosclerosis evaluation

Carotid intima-media thickness (cIMT) was utilized as the structural proxy for subclinical atherosclerosis. High-resolution B-mode ultrasonography was conducted using a GE Healthcare LOGIQ E9 system equipped with a GE 9L linear array transducer operating at a maximum frequency of 10 MHz. A single, highly experienced radiologist, fully blinded to all corresponding clinical and laboratory parameters, performed all baseline and 6-month examinations to avoid inter-observer bias. Patients were scanned in the supine position with the neck subtly extended. cIMT was defined as the distance separating the lumen-intima interface from the leading edge of the media-adventitia interface on the far wall of the common carotid artery. Measurements were logged at a predefined, standardized segment proximal to the carotid bifurcation and reported in millimeters (mm) [12].

### Statistical analysis

No formal a priori sample size calculation was performed because this was an exploratory prospective observational study based on eligible patients initiating biologic therapy during the predefined study period. Statistical analysis was performed using IBM SPSS Statistics (version 32.0; IBM Corp., Armonk, NY, USA). Continuous variables were tested for normality using the Shapiro–Wilk test. Normally distributed variables were expressed as mean ± standard deviation (SD), whereas non-normally distributed variables were presented as median (Min–Max). Comparisons between baseline and 6-month follow-up measurements were performed using paired-samples t-tests for normally distributed variables and Wilcoxon signed-rank tests for non-normally distributed variables. Comparisons between anti-TNF and non-anti-TNF treatment groups were conducted using independent-samples t-tests. Associations between continuous variables were assessed using correlation analysis. To identify independent predictors of changes in CitH3 levels (ΔCitH3), multivariable linear regression analysis was performed with ΔCitH3 as the dependent variable and selected clinical and laboratory variables as independent predictors. Multicollinearity was assessed using variance inflation factor (VIF) and tolerance statistics. All tests were two-tailed, and a *p*-value < 0.05 was considered statistically significant.

## Results

### Patient characteristics and effects of biologic therapy

Thirty-six patients with RA initiating biologic therapy were included in the study. The mean age was 58.7 ± 11.5 years, and 77.8% of participants were female. Rheumatoid factor and anti-citrullinated protein antibody positivity were observed in 83.3% and 66.6% of patients, respectively. Twenty-three patients (63.9%) received anti-TNF agents, whereas thirteen (36.1%) received non-anti-TNF biologic therapies. No adverse events, biologic dose modifications, or treatment switches were reported during the 6-month follow-up period. Baseline demographic and clinical characteristics are presented in Table 1.

After 6 months of biologic treatment, statistically significant improvements were observed in inflammatory activity and disease severity. Median CRP levels decreased from 6.0 to 3.0 mg/L (*p* < 0.001), while median ESR decreased from 27.5 to 13.5 mm/h (*p* < 0.001). Similarly, median DAS28 declined significantly from 3.59 to 2.97 (*p* < 0.001).

Regarding lipid parameters, there was a statistically significant increase in HDL-C levels from 56.36 ± 16.21 to 67.47 ± 17.56 mg/dL (*p* < 0.001), whereas no significant changes were observed in total cholesterol, triglycerides, or LDL-C levels.

A statistically significant reduction in carotid intima-media thickness was observed following biologic therapy, with median cIMT decreasing from 1.00 mm at baseline to 0.75 mm after 6 months (*p* < 0.001). Circulating CitH3 levels, used as a marker of NETs, were also presented with a statistically significant reduction, decreasing from a median of 3.89 to 2.56 ng/mL (*p* = 0.025) (Table 1).

### Baseline determinants of CitH3

Multivariable linear regression analysis was performed to identify factors independently associated with baseline CitH3 levels. Among the variables included in the model, ESR emerged as the only significant predictor of CitH3 concentrations (B = 0.157, β = 0.448, *p* = 0.018). In contrast, CRP (*p* = 0.907), HDL (*p* = 0.176), and cIMT (*p* = 0.835) were not significantly associated with CitH3 levels (Table 2).

**Table 2:** Multivariable regression analysis of baseline CitH3 levels

Variable	B	β	*p*-value	95% CI
CRP	0.013	0.022	0.907	−0.205 to 0.230
ESR	0.157	0.448	0.018	0.028 to 0.285
HDL	−0.077	−0.223	0.176	−0.191 to 0.037
IMT	0.915	0.035	0.835	−7.970 to 9.800

CitH3 was used as the dependent variable. Values represent unstandardized (B) and standardized (β) regression coefficients from multivariable linear regression analysis. CRP, C-reactive protein; ESR, erythrocyte sedimentation rate; HDL, high-density lipoprotein cholesterol; cIMT, carotid intima-media thickness

### Determinants of treatment-induced changes in CitH3

To investigate factors associated with biologic therapy-induced changes in NET release, a multivariable regression model was constructed using ΔCitH3 as the dependent variable. The model was statistically significant (R^2^ = 0.330, adjusted R^2^ = 0.244, F = 3.818, *p* = 0.012).

Within this model, ΔHDL was the only independent predictor of ΔCitH3 (B =  − 0.346, β =  − 0.505, *p* = 0.003). Neither ΔDAS28 (*p* = 0.199), ΔCRP (*p* = 0.995), nor ΔcIMT (*p* = 0.976) demonstrated statistically significant associations with changes in CitH3 levels (Table 3).

**Table 3:** Multivariable regression analysis of ΔCitH3

Variable	B	β	*p*-value	95% CI
ΔIMT	0.250	0.005	0.976	−16.646 to 17.145
ΔDAS28	2.352	0.230	0.199	−1.299 to 6.003
ΔHDL	−0.346	−0.505	0.003	−0.563 to −0.128
ΔCRP	−0.001	−0.001	0.995	−0.210 to 0.209

ΔCitH3 was used as the dependent variable. Values represent unstandardized regression coefficients (B), standardized regression coefficients (β), and corresponding *p*-values derived from multivariable linear regression analysis. Independent variables included ΔDAS28, ΔCRP, ΔHDL cholesterol, and ΔcIMT. The overall model was statistically significant (R^2^ = 0.330, adjusted R^2^ = 0.244, F = 3.818, *p* = 0.012). Δ denotes the change between baseline and 6-month follow-up. CRP, C-reactive protein; HDL, high-density lipoprotein cholesterol; DAS28, Disease Activity Score in 28 joints; cIMT, carotid intima-media thickness. Statistical significance was defined as *p* < 0.05

### Comparison between anti-TNF and non-anti-TNF biologic therapies

Patients treated with anti-TNF agents exhibited significantly higher baseline ESR values compared with those receiving non-anti-TNF biologics (36.5 ± 17.9 vs. 25.6 ± 9.4 mm/h, *p* = 0.023).

No statistically significant differences were observed between treatment groups regarding baseline CitH3 concentrations (*p* = 0.420) or HDL levels (*p* = 0.481). Furthermore, treatment-induced changes in CitH3 (*p* = 0.870) and HDL (*p* = 0.091) were comparable between anti-TNF and non-anti-TNF therapies (Table 4).

**Table 4:** Anti-TNF versus non-anti-TNF biologic therapy

Variable	Non-anti-TNF (*n* = 13)	Anti-TNF (*n* = 23)	*p*-value
ESR (baseline)	25.6 ± 9.4	36.5 ± 17.9	0.023
CitH3 (baseline)	3.76 ± 3.28	5.11 ± 6.62	0.420
HDL (baseline)	59.1 ± 18.0	54.8 ± 15.3	0.481
ΔCitH3	−1.73 ± 2.82	−1.99 ± 6.46	0.870
ΔHDL	−0.68 ± 5.59	−0.59 ± 8.66	0.091

Data are presented as mean ± SD. Comparisons between treatment groups were performed using independent samples t-tests. Δ denotes the change between baseline and 6-month follow-up. ESR, erythrocyte sedimentation rate; HDL, high-density lipoprotein cholesterol; CitH3, citrullinated histone H3. Statistical significance was defined as *p* < 0.05

## Discussion

The present prospective study found that, over six months following initiation of biologic therapy, patients with RA experienced significant improvements in disease activity, systemic inflammation, lipid profile, circulating CitH3 levels, and subclinical atherosclerosis. Among the variables examined, changes in HDL cholesterol were independently associated with changes in CitH3, whereas changes in disease activity, CRP, ESR, oxLDL, and cIMT were not independently associated with ΔCitH3. These findings suggest a potential relationship between changes in lipid metabolism and NET-associated activity during biologic treatment. However, given the single-arm observational design, the absence of an untreated comparator group, and the relatively small sample size, these temporal associations should be interpreted as exploratory and hypothesis-generating rather than as evidence of a causal treatment effect.

The role of NETs in the pathogenesis of RA has attracted increasing attention over the past decade. NETs contribute to synovial inflammation through the release of citrullinated autoantigens, proteolytic enzymes, and pro-inflammatory mediators, thereby amplifying both innate and adaptive immune responses [15]. Elevated circulating levels of NET-associated products, including citrullinated histones, have been consistently reported in patients with active RA and are associated with increased disease activity and inflammatory burden [16, 17]. Consistent with these observations, our baseline regression analysis identified ESR as the sole independent predictor of circulating CitH3 levels, further supporting the close link between NETs and disease activity. Given the sample size, however, this finding should be regarded as exploratory.

Beyond joint inflammation, NETs have emerged as a potential mediator of accelerated cardiovascular disease in RA. NET-derived molecules promote endothelial dysfunction, oxidative stress, leukocyte recruitment, and plaque instability, thereby contributing to the progression of atherosclerosis [18–20]. This mechanistic link provides a biological rationale for investigating whether modulation of NET-associated activity contributes to the vascular effects observed during effective treatment of RA. However, the present study cannot establish such a mechanistic or causal relationship. Given the well-established excess cardiovascular burden in RA patients, these observations may provide an additional biological explanation for the cardiovascular benefits associated with effective control of systemic inflammation [21, 22].

Consistent with the proposed role of NETs in RA pathophysiology, biologic therapy was associated with a significant reduction in circulating CitH3 levels, consistent with reduced NET-associated activity. A significant reduction in cIMT was also observed, suggesting improvement in subclinical vascular involvement. Despite these parallel changes, no independent association was identified between changes in CitH3 and cIMT. Although experimental evidence supports a mechanistic role of NETs in atherosclerosis through endothelial dysfunction, oxidative stress, and vascular inflammation [18, 20], this finding should be interpreted with caution. CitH3 reflects dynamic neutrophil activation, whereas cIMT is a structural marker of cumulative vascular injury influenced by multiple cardiovascular determinants. Therefore, the lack of a short-term association may simply reflect the different biological kinetics of these markers rather than the absence of a mechanistic link. Furthermore, although the magnitude of cIMT reduction over six months appears substantial, similar improvements have been reported following effective biologic therapy in RA, particularly when vascular assessments are performed using standardized imaging protocols [23, 24].

One of the most notable findings of the present study was the independent association between changes in HDL cholesterol and changes in CitH3 levels. This observation is of particular interest given the increasingly recognized role of HDL beyond lipid transport and cardiovascular protection. Although HDL has traditionally been regarded as an anti-atherogenic lipoprotein because of its central role in reverse cholesterol transport, accumulating evidence indicates that HDL also exerts important anti-inflammatory, antioxidant, and immunomodulatory effects [25, 26]. The independent inverse association between changes in HDL cholesterol and CitH3 levels may reflect a fundamental immunometabolic link centered on neutrophil cholesterol homeostasis. Functional HDL promotes cholesterol efflux via the ATP-Binding Cassette Transporters A1 and G1 (ABCA1 and ABCG1); clinical and experimental data suggest that lipid accumulation within neutrophil membranes lowers the threshold for NET formation by enhancing Toll-Like Receptor (TLR) signaling and Nicotinamide Adenine Dinucleotide Phosphate (NADPH) oxidase activity. Therefore, the recovery of HDL levels might restore the cholesterol efflux capacity, leading to membrane stabilization and reduced activation of peptidylarginine deiminase 4 (PAD4), a key enzyme driving chromatin decondensation through histone citrullination. Furthermore, HDL-associated proteins, such as Paraoxonase-1 (PON1), may further suppress NET formation by neutralizing oxidative signals required for histone citrullination.

Our findings suggest that the quantitative gain in HDL cholesterol under bDMARD therapy is not merely a marker of reduced systemic inflammation but may also reflect a functional restoration of the lipoprotein’s ability to dampen neutrophil-driven vascular interference [27, 28]. Such a biologically relevant interaction between lipid metabolism and NETs supports the concept that alterations in lipoprotein function may contribute to the regulation of inflammatory pathways in RA [24, 29–34].

Interestingly, no significant relationship between HDL and CitH3 levels was observed at baseline. The association became evident only when treatment-induced changes were examined longitudinally. This observation raises the possibility that the relationship between HDL and NETs may be better captured through dynamic changes rather than static cross-sectional measurements. In other words, patients experiencing the greatest improvement in HDL levels were also those demonstrating the greatest reduction in NETs activity.

Another noteworthy finding was the absence of significant differences between anti-TNF and non-anti-TNF biologic therapies in baseline CitH3 levels or treatment-associated changes in CitH3. Although patients receiving anti-TNF agents exhibited higher baseline ESR values, both therapeutic strategies appeared to exert comparable effects on NETs suppression. This finding is biologically plausible: IL-6 blockade with tocilizumab has been shown to attenuate neutrophil activation and reduce surrogate markers of NETs in inflammatory settings, potentially via dampening IL-6-driven priming of granulopoiesis and endothelial activation [35–37]. Conversely, TNF inhibition may indirectly suppress NETs by reducing systemic inflammatory tone and oxidative signals that facilitate chromatin decondensation, with studies reporting reductions in MPO–DNA complexes or PAD4-dependent signaling under TNF blockade in experimental and clinical contexts [38–40]. Taken together, these data support the notion that effective control of upstream inflammatory pathways—rather than the specific cytokine target may be the principal determinant of NET reduction. Nonetheless, our sample size was relatively small and enriched for tocilizumab among non-anti-TNF agents, limiting the statistical power to detect class-specific differences; adequately powered, head-to-head studies using standardized NET endpoints such as CitH3, MPO–DNA complexes, and functional NET assays, are warranted to clarify potential mechanistic differences between IL-6 and TNF pathway inhibition.

Several strengths should be noted. The prospective design with paired assessments at baseline and six months allowed longitudinal evaluation of changes in circulating CitH3, inflammatory and lipid parameters, and cIMT within the same patients. All participants were evaluated using a standardized study protocol, and cIMT measurements were performed by a single experienced radiologist, blinded to the clinical and laboratory data, thereby reducing potential inter-observer variability. In addition, the simultaneous assessment of NET-associated activity, systemic inflammation, lipid profile, and subclinical atherosclerosis allowed exploration of potential relationships between these parameters during biologic treatment. Finally,the longitudinal analysis provided an opportunity to examine treatment-associated changes rather than relying solely on cross-sectional associations.

This study has several limitations. Its single-center, single-arm observational design, small sample size, absence of a formal a priori sample size calculation, and short six-month follow-up limit statistical power, causal inference, and generalizability. In particular, the absence of an untreated or comparator group prevents determination of whether the observed temporal changes were attributable specifically to biologic therapy, as opposed to regression to the mean, temporal variation, or other concomitant changes. The relatively small sample size also limits the stability of multivariable regression estimates and substantially reduces the power of the anti-TNF versus non-anti-TNF subgroup comparisons; consequently, these analyses should be considered exploratory. The predominance of tocilizumab in the non-anti-TNF group may have reduced the ability to detect class-specific effects and long-term relationships between NETs and vascular remodeling. In addition, NET formation was assessed using only one surrogate marker (CitH3), without complementary functional assays, while background csDMARDs and glucocorticoids may have influenced the observed effects. Finally, the association between HDL and CitH3 should be interpreted as hypothesis-generating rather than causal and requires confirmation in larger, multicenter prospective studies.

## Conclusion

In this small prospective observational cohort, initiation of biologic therapy was associated with a significant reduction in circulating CitH3 levels, together with improvements in disease activity, systemic inflammation, HDL cholesterol, and cIMT. The independent longitudinal association between increased HDL cholesterol and reduced CitH3 levels raises the possibility of an immunometabolic relationship between HDL and NET-associated activity in RA. However, given the single-arm design, small sample size, short follow-up, and use of CitH3 as a single surrogate marker of NET-associated activity, these findings should be considered exploratory and hypothesis-generating. Larger, controlled prospective studies incorporating complementary NET biomarkers and functional assays are warranted to confirm these observations and clarify their clinical significance.

## Supplementary Information

Below is the link to the electronic supplementary material.

Supplementary file1 (DOCX 46 KB)

## Data Availability

The datasets generated and/or analyzed during the current study are available from the corresponding author upon reasonable request and subject to the appropriate ethical and data protection requirements.
