# Supplementary material for: Neutrophil extracellular trap suppression in rheumatoid arthritis patients treated with biologic therapy: a prospective observational study
Source: Clin Rheumatol. 2026 Oct 3;45(11):6573–81. doi: 10.1007/s10067-026-08454-8 (PMC13723562; doi:10.1007/s10067-026-08454-8)
Supplement: Supplementary file 1 [file 10067_2026_8454_MOESM1_ESM.docx]

STROBE Statement—Checklist of items that should be included in reports of ***cohort studies***

|  | Item No | Recommendation | Page No |
| --- | --- | --- | --- |
| **Title and abstract** | 1 | (*a*) Indicate the study’s design with a commonly used term in the title or the abstract | Title / Abstract page No1 |
|  |  | (*b*) Provide in the abstract an informative and balanced summary of what was done and what was found | Abstract page No 1-2 |
| Introduction | | | |
| Background/rationale | 2 | Explain the scientific background and rationale for the investigation being reported | Introduction page No 4 |
| Objectives | 3 | State specific objectives, including any prespecified hypotheses | Introduction page No 4 |
| Methods | | | |
| Study design | 4 | Present key elements of study design early in the paper | Methods – Study design and participants  Page No 4 |
| Setting | 5 | Describe the setting, locations, and relevant dates, including periods of recruitment, exposure, follow-up, and data collection | Methods – Study design and participants  Page No 4 |
| Participants | 6 | (*a*) Give the eligibility criteria, and the sources and methods of selection of participants. Describe methods of follow-up | Methods – Study design and participants  Page No 4 |
|  |  | (*b*) For matched studies, give matching criteria and number of exposed and unexposed | Not Available |
| Variables | 7 | Clearly define all outcomes, exposures, predictors, potential confounders, and effect modifiers. Give diagnostic criteria, if applicable | Methods – Study design and participants; Laboratory assessments; cIMT assessment  Page No 5 |
| Data sources/ measurement | 8* | For each variable of interest, give sources of data and details of methods of assessment (measurement). Describe comparability of assessment methods if there is more than one group | Methods – Study design and participants; Laboratory assessments; cIMT assessment  Page No 5 |
| Bias | 9 | Describe any efforts to address potential sources of bias | Methods – Study design and participants; cIMT assessment Page No 5 |
| Study size | 10 | Explain how the study size was arrived at | Methods – Statistical analysis  Page No 5-6 |
| Quantitative variables | 11 | Explain how quantitative variables were handled in the analyses. If applicable, describe which groupings were chosen and why | Methods – Statistical analysis  Page No 5-6 |
| Statistical methods | 12 | (*a*) Describe all statistical methods, including those used to control for confounding | (a)Methods – Statistical analysis  Page No 5-6  (b) Results – Anti-TNF vs non-anti-TNF analysis  (c) Methods – Statistical analysis |
|  |  | (*b*) Describe any methods used to examine subgroups and interactions | (d) Methods – Study design and participants / Results page No 5  (e) Not Available |
|  |  | (*c*) Explain how missing data were addressed |  |
|  |  | (*d*) If applicable, explain how loss to follow-up was addressed |  |
|  |  | (*e*) Describe any sensitivity analyses |  |
| Results | | |  |
| Participants | 13* | (a) Report numbers of individuals at each stage of study—eg numbers potentially eligible, examined for eligibility, confirmed eligible, included in the study, completing follow-up, and analysed | (a) Results – Study population page No 5-6  (b) Results – Study population  (c) Not Available |
|  |  | (b) Give reasons for non-participation at each stage |  |
|  |  | (c) Consider use of a flow diagram |  |
| Descriptive data | 14* | (a) Give characteristics of study participants (eg demographic, clinical, social) and information on exposures and potential confounders | (a) Table 1 (b) Not available (c) Methods – Study design and participants; Results page No 5-6 |
|  |  | (b) Indicate number of participants with missing data for each variable of interest |  |
|  |  | (c) Summarise follow-up time (eg, average and total amount) |  |
| Outcome data | 15* | Report numbers of outcome events or summary measures over time | Table 1; Results |

| Main results | 16 | (*a*) Give unadjusted estimates and, if applicable, confounder-adjusted estimates and their precision (eg, 95% confidence interval). Make clear which confounders were adjusted for and why they were included | (a)Results – Main results; Tables 2 and 3 page No6  (b)Not available  (c) Not available |
| --- | --- | --- | --- |
|  |  | (*b*) Report category boundaries when continuous variables were categorized |  |
|  |  | (*c*) If relevant, consider translating estimates of relative risk into absolute risk for a meaningful time period |  |
| Other analyses | 17 | Report other analyses done—eg analyses of subgroups and interactions, and sensitivity analyses | Results – Other analyses; Table 4 page No 6 |
| Discussion | | | |
| Key results | 18 | Summarise key results with reference to study objectives | Discussion – Key results page No 7,8 |
| Limitations | 19 | Discuss limitations of the study, taking into account sources of potential bias or imprecision. Discuss both direction and magnitude of any potential bias | Discussion – Limitations page No 8 |
| Interpretation | 20 | Give a cautious overall interpretation of results considering objectives, limitations, multiplicity of analyses, results from similar studies, and other relevant evidence | Discussion – Interpretation page No7 |
| Generalisability | 21 | Discuss the generalisability (external validity) of the study results | Discussion – Generalisability page No 8,9 |
| Other information | | | |
| Funding | 22 | Give the source of funding and the role of the funders for the present study and, if applicable, for the original study on which the present article is based | Declarations – Funding page No 10 |

*Give information separately for exposed and unexposed groups.

**Note:** An Explanation and Elaboration article discusses each checklist item and gives methodological background and published examples of transparent reporting. The STROBE checklist is best used in conjunction with this article (freely available on the Web sites of PLoS Medicine at http://www.plosmedicine.org/, Annals of Internal Medicine at http://www.annals.org/, and Epidemiology at http://www.epidem.com/). Information on the STROBE Initiative is available at http://www.strobe-statement.org.
